# Does bright  light have an anxiolytic effect? - an open trial

**DOI:** 10.1186/1471-244X-7-62

**Published:** 2007-10-30

**Authors:** Shawn D Youngstedt, Daniel F Kripke

**Affiliations:** 1 Department of Exercise Science, Norman J. Arnold School of Public Health University of South Carolina, Columbia, SC 29208 USA; 2 Department of Psychiatry, Dorn VA Medical Center, Columbia, South Carolina, 29209, USA; 3 Department of Psychiatry and Sam and Rose Stein Institute for Research on Aging, University of California, San Diego, La Jolla, CA 92093-0667, USA

## Abstract

**Background:**

The aim of this open trial was to examine the influence of acute bright light exposure on anxiety in older and young adults.

**Methods:**

This study was ancillary to a complex 5-day laboratory experiment testing phase-responses to light at all times of the day. On 3 consecutive days, participants were exposed to bright light (3,000 lux) for 3 hours. The Spielberger State-Trait Anxiety Inventory (Form Y1) was administered 5 minutes before and 20 minutes after each treatment. Mean state anxiety before and after treatment were analyzed by age, sex, and time ANOVA. To avoid floor effects, only participants with baseline STAI levels of ≥ 25 were included.

**Results:**

A significant anxiolytic effect of bright light was found for the mean data, as well as for each of the three days. No significant main effect of age, sex, or interaction of these factors with STAI change were found.

**Conclusion:**

The results show consistent and significant (albeit modest) anxiolytic effects following acute bright light exposure in low anxiety adults. Further randomized, controlled trials in clinically anxious individuals are needed.

## Background

Anxiety is the most common mental illness in the US [1,2]. Moreover, complaints of anxiety are common among healthy individuals and have been associated with numerous negative health consequences [3-5], absenteeism [6], and decreased work productivity [6,7].

Anxiolytic drugs have limited long-term efficacy. Their adverse side effects include dependency (for benzodiazepine agonists), drowsiness [8], impaired cognition and memory [8-10], and sexual dysfunction [10,11]. Alternative or adjuvant treatments for anxiety might be valuable.

Bright light exposure would be a potentially attractive anxiolytic treatment. The antidepressant effects of bright light are well-established for winter depression [12,13], as well as many other types of nonseasonal depression [14-17]. The common co-morbidity [5,18] and neurochemical similarity between anxiety and depression (e.g., responsiveness to the same drugs) [19] provide rationales for expecting that bright light might also have substantial anxiolytic effects.

Although there has been little systematic investigation of the anxiolytic effect of bright light, there is some experimental support for this effect. For example, in research of light treatment for winter depression, anxiety-related symptoms have been reduced to approximately the same degree as depressive symptoms [20]. Similar reduction in anxiety and depression symptoms following light exposure have also been noted in healthy individuals and individuals with subsynromal SAD [21]. Some evidence suggests that both anxiolytic and antidepressant effects of light might be mediated by serotonergic mechanisms [22]. The primary aim of the present study was to explore the anxiolytic effect of bright light in a large sample. Secondary aims were to contrast effects in older (ages 60–75 yrs) vs. young (ages 18–30 yrs) adults and in women vs. men. The study was ancillary to a complex 5–6 day laboratory protocol, which allowed us to examine this effect on 3 consecutive days. The sample generally had low levels of depression, which allowed exploration of anxiolytic effects independent of antidepressant effects of bright light.

## Methods

Older adults ages 60–75 yr (66.6 ± 4.5 yr) and young adults ages 18–30 yrs (23.4 ± 3.8 yr) were recruited by word of mouth, referrals, announcements in local media, and advertising in newspapers, adult fitness centers, and senior pages. Initial inclusion criteria, based on several screening questionnaires, required good health and regular participation in vigorous aerobic exercise. Exclusions included: (1) having > 1 of the major risk factors for coronary artery disease [23] including family history of early myocardial infarction, current smoking, hypertension, history of hypercholesterolemia, and diabetes mellitus; (2) having any major symptoms or signs of cardiopulmonary disease [23], including chest pain, dizziness or syncope, orthopnea or nocturnal dyspnea, ankle edema, heart papitations, claudication, serious heart murmur, and excessive shortness of breath; (3) recent shift-work (previous 2 months) or travel across multiple time zones (previous 4 weeks); (4) abnormal sleep-wake schedule; (5) depression; (6) use of alpha or beta blockers, antihypertensives, or antidepressants; (7) or any health or mental condition that would contraindicate participating in the rigors of the experiment.

Prior to participation in the study, written informed consent was obtained from each participant, as approved by the UCSD Institutional Review Board. Included in the consent was a description that the study would include assessment of the influence of bright light on mood, which is the focus of the present report. The research was conducted in compliance with the Helsinki Declaration.

Once recruited, participants were required to pass a medical interview and further medical screening. Exclusions included elevations in fasting plasma lipids and glucose, hypertension, abnormal resting 12-lead EKG, and signs of heart disease during a physician-supervised treadmill test to volitional exhaustion. Participants were not given a psychiatric interview.

### Laboratory protocol

The 5–6 day laboratory protocol was designed to examine the influence of bright light or exercise on the circadian system. Participants were randomized to bright light or exercise treatment, but the present report is limited to data associated with the bright light treatment. Each participant stayed in a studio-apartment room and followed a 90 minute "ultra-short" sleep wake cycle [24], involving 60 min wake intervals in dim light (< 50 lux) and 30 min sleep intervals in darkness. This cycle was repeated around-the-clock throughout the 5-day period. Food and drink were available *ad libitum *(excluding caffeine and alcohol). The protocol included around-the-clock collection of urinary and saliva samples (at 90 min intervals) and completion of several visual analogue scales at 4-hr intervals. Tests of retinal circadian rhythms (e.g., electrooculography, visual threshold) were performed around the clock on a subset of participants (n = 12). During the wake periods, participants were free to engage in sedentary activities, including watching television, reading, receiving visitors, etc. Strenuous exercise was prohibited. Participants completed the Center for Epidemiological Studies Depression Scale (CESD) [25] on the first and last days of the laboratory study.

### Light treatments

Participants were exposed to 3 hr of bright light (3,000 lux) on 3 consecutive days. The treatment was administered via overhead cool-white fluorescent lights which distributed the light levels evenly throughout the room. The light was administered at one of 8 randomly assigned times-of-day or night, but was provided at the same time-of-day or night across the 3 days for each individual. The treatments were administered in a phase-response curve experiment establishing the direction and magnitude of phase-shift depending upon the time of treatment.

### Anxiety assessement

Anxiety was assessed with Spielberger's State-Trait Anxiety Inventory (STAI; Form Y1, i.e., "how you feel at the moment") [26] at 5 min before and 20 min following all three light treatments. The reliability and validity of the STAI are well established [26]. Participants were given standardized instructions prior to completing the STAI questionnaire.

### Data analysis

To avoid floor effects, an *a priori *decision was made to exclude participants for whom baseline STAI were < 25, which would be approximately one standard deviation below population norms [26]. Data were averaged across the 3 days of assessment, and analyzed by repeated measures ANOVA, comparing responses by age group and sex. In addition, ANOVAs were calculated for each of the 3 individual days of assessment. Normal distribution of the data was established. Effect size was calculated by subtracting the mean post-light STAI from the mean pre-light STAI and dividing this difference by the pooled standard deviation. Since analysis indicated that there was no significant influence of time-of-day on baseline STAI levels or on anxiolytic responses to bright light, data were collapsed across all times of testing in the present report. Comparison of anxiolytic response between individuals whose baseline STAI levels were within the range found for clinical populations (= 48) [26] vs. individuals with lower STAI levels was made via an independent t-test. Normal distribution of the data was verified.

## Results

The number of participants excluded from analysis due to low baseline STAI levels was 25. The present analyses included 79 participants out of a total of 104. Four participants had clinical levels of baseline STAI (50.8 ± 0.87) (though none had formal DSM IV diagnoses of anxiety disorders), whereas 75 participants had lower levels (31.0 ± 0.57).

The older participants (n = 33) were ages 59–75 years (66.6 ± 4.5 yr) and the young participants (n = 46) were ages 18–30 yr (23.4 ± 3.8 yr). Both age groups had low levels of depressed mood (CESD levels [25] of 8.6 ± 1.0 and 6.9 ± 1.0, respectively, for the young and older participants). No significant mood difference between age groups was found in laboratory levels of CES-D.

A significant mean post-treatment reduction in STAI was found [F(1,78) = 17.92, p < 0.001]. The corresponding effect size was 0.36 (95% CI: 0.19–0.53). The reduction in STAI was significantly greater for participants with clinical levels of STAI (reduction of 12.37 ± 6.880 compared with those with lower STAI levels (1.8 ± 0.40) [t(1,770 = 4.76, p <v0.001]. The anxiolytic effect was also observed separately for each of the three DAYS (p = 0.04, 0.02, < 0.001, respectively). The corresponding effect sizes (and 95% CI) were 0.23 (0.01–0.45), 0.20 (0.02–0.38), and 0.38 (0.22–0.54), respectively.

**Figure 1:**
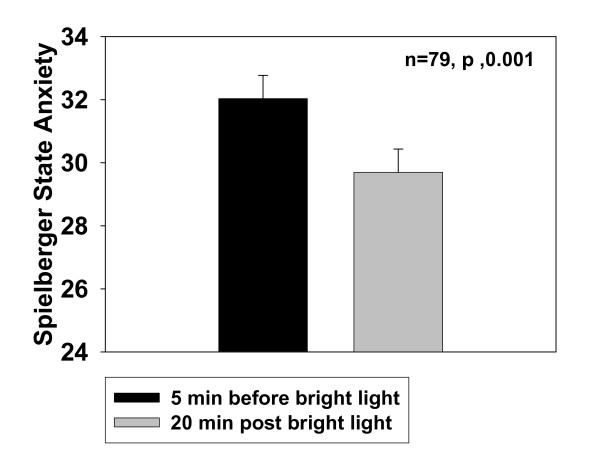
Mean (± SE) state anxiety (STAI-Y1) assessed 5 min before and 20 min after bright light treatment (3 hr at 3,000 lux) averaged over three consecutive days. Data are combined across age and sex.

There was no significant age or sex effect. STAI data, collapsed across sex and age group are displayed in Figure 1.

## Discussion

The results indicate a significant anxiolytic effect of acute bright light exposure. Moreover, the effect was consistent for each of the three days. The anxiolytic effect did not vary significantly by age group or sex of the participants. The corresponding effect size is considered small [27], but comparable to other anxiolytic stimuli in low anxious adults [28,29].

The data are consistent with other research suggesting a reduction of anxiety symptoms following bright light treatment of winter depression [20]. A unique aspect of the present study is that the anxiolytic effects were observed after acute exposure to bright light, whereas others reports had considered effects after 2 or more weeks of light treatment.

The results are not consistent with a recent study by Goel and Etwaroo [30], who found no significant effect of acute bright light treatment on the POMS-tension subscale, but a significant antidepressant effect was observed. The discrepancy in findings might be attributed to methodological differences. Whereas the present study involved 3 hr of exposure to 3,000 lux light and assessment of anxiety change at 20 min post-exposure, the Goel and Etwaroo study examined anxiety changes during exposure to 30 min of 10,000 lux light [30].

There were several noteworthy limitations of the present study. First, an obvious methodological limitation was the lack of a control group, which was not possible in this ancillary open trial. Nonetheless, we do not think that the observed changes in STAI can be readily attributed to behavioral artifacts, such as demand characteristics or expectancy effects. Anecdotally, it seemed that the subjects viewed completion of the STAI as a small part of a complex study. As much focus was placed on following the ultra-short sleep-wake schedule, collection of urine samples, etc., it seemed that subjects were unaware of the research hypothesis of this ancillary study. Were demand or expectancy effects operating, greater anxiolytic effects might be expected. The STAI changes are likely also not attributable to experimentally imposed "time-out" from daily stressors, as the bright light treatment was not administered until the participants were in the laboratory for = 30 hr. Nonetheless, controlled, randomized studies are needed to verify these findings.

A second limitation was the low baseline levels of anxiety of the participants. As expected, the few high-anxious participants had far greater anxiolytic responses than the other participants. Despite receiving recommended standardized instructions for completing the STAI, a remarkably high number of participants were excluded from the analyses due to low baseline STAI levels (< 25). The decision to exclude these individuals was made *a priori*. The reasons for the low baseline STAI levels are unclear. The participants might have made efforts to complete the STAI questionnaires in a socially desirable manner. However, similarly abnormally low values were not observed for the CES-D data. The lack of inclusion of individuals ages 31–58 yrs, who tend to have high rates of anxiety, might have contributed to low baseline levels.

A third limitation was that the high levels of health and fitness of the participants was not representative of the population, particularly not that of older individuals. However, this limitation might have led to an underestimation of the efficacy of bright light, which have been more clearly demonstrated in less healthy individuals. Some correlates of health and fitness, such as better psychological health and greater habitual exposure to bright outdoor light, might have attenuated the anxiolytic effects of bright light.

A fourth limitation might have been the unique laboratory environment for exploring psychological benefits of bright light, involving prolonged maintenance of the ultra-short sleep-wake cycle, a moderate degree of sleep deprivation, low levels of light exposure, etc. However, despite the rigors of the experiment, it did not appear to be "stressful". For example there were no changes in pre-treatment STAI levels between DAYs 1–3. Indeed, many subjects reported that the experience was calming. As addressed above, by separating the subjects from their usual daily stressors, the environment might have resulted in an underestimation of the anxiolytic effects of bright light treatment.

That participants had generally had low levels of depression suggests that the anxiolytic effect of light exposure was independent of significant antidepressant effects. Moreover, in post-hoc analysis, we found no significant correlation of anxiolytic effect with baseline level of depression (r = 0.043).

## Conclusion

Notwithstanding its limitations, the present study provides provocative data addressing an important, but neglected area of research. Although anxiety and depression are the most common mental disorders, far more research focus has been placed on depression. This limitation is certainly true for bright light treatment. Whereas the antidepressant effects of bright light have been examined in dozens of studies, the studies did not focus on potential anxiolytic effects of bright light. That significant anxiolytic effects were observed following acute exposure in low-anxious subjects gives rise to the exciting possibility that far greater effects of bright light might be observed following chronic treatment in high-anxious individuals. Further randomized, controlled experiments are needed.

## Competing interests

The author(s) declare that they have no competing interests.

## Authors' contributions

SDY conceived the idea, directed collection of the data, and drafted the manuscript. DFK helped interpret the results and write the manuscript. All authors read and approved the manuscript.

## Pre-publication history

The pre-publication history for this paper can be accessed here:


